# Semi-automated analysis of digital whole slides from humanized lung-cancer xenograft models for checkpoint inhibitor response prediction

**DOI:** 10.18632/oncotarget.27069

**Published:** 2019-07-16

**Authors:** Daniel Bug, Friedrich Feuerhake, Eva Oswald, Julia Schüler, Dorit Merhof

**Affiliations:** ^1^Institute of Imaging and Computer Vision, RWTH-Aachen University, D-52074 Aachen, Germany; ^2^Institute for Pathology, Hannover Medical School, D-30625 Hannover, Germany; ^3^Institute for Neuropathology, University Clinic Freiburg, D-79106 Freiburg im Breisgau, Germany; ^4^Charles River Discovery, Research Services Germany GmbH, D-79108 Freiburg im Breisgau, Germany

**Keywords:** deep learning, digital pathology, histology, non small cell lung cancer, xenograft

## Abstract

We propose a deep learning workflow for the classification of hematoxylin and eosin stained histological whole-slide images of non-small-cell lung cancer. The workflow includes automatic extraction of meta-features for the characterization of the tumor. We show that the tissue-classification produces state-of-the-art results with an average F1-score of 83%. Manual supervision indicates that experts, in practice, accept a far higher percentage of predictions. Furthermore, the extracted meta-features are validated via visualization revealing relevant biomedical relations between the different tissue classes. In a hypothetical decision-support scenario, these meta-features can be used to discriminate the tumor response with regard to available treatment options with an estimated accuracy of 84%. This workflow supports large-scale analysis of tissue obtained in preclinical animal experiments, enables reproducible quantification of tissue classes and immune system markers, and paves the way towards discovery of novel features predicting response in translational immune-oncology research.

## INTRODUCTION

Digital Pathology is a rapidly emerging field introducing modern image processing, computational analysis and machine learning algorithms to pathological workflows. In this context, whole-slide scanners are utilized to digitize microscopy images of stained histological tissue, resulting in gigapixel-sized images. State-of-the-art deep learning methods implemented for Graphics Processing Units use provide the necessary computational capacity to process the immense amount of data. From a biomedical perspective, a detailed analysis of tissue distribution and co-localization contributes objective and reproducible measures to characterize the tumor-micro-environment (TME). Quantifying these features is of particular interest in pre-clinical as well as clinical research in the field of immune-oncology. Although numerous clinical trials are ongoing and the effort in pre-clinical drug development is tremendous in academia as well as industry, only a small proportion of patients benefits from these innovative treatment strategies. The Single-Mouse-Trial (SMT) [1, 2] using patient derived xenografts (PDX) in humanized mice is a highly predictive screening approach for pre-clinical immune-oncology drug development. In this work, we provide an exceedingly automated analysis of whole-slide images from PDX models acting as a support system to categorize the tumor behavior and host immune system. In particular, we compare the tumor and immune system interaction under treatment with anti-PD-L1, anti-CTLA4, and a combination thereof versus the untreated PDX model. As we consider a screening application, the amount of data on the technical side requires a semantic annotation framework, which aims at predicting output images of input size, and thereby drastically reduces redundancy compared to patchwise classification pipelines. Furthermore, a semantic scenario inherently considers a large pixel context, which is certainly relevant in histological tissue classification. Realizations of semantic annotations are found in the fully-convolutional network (FCN) for semantic segmentation [3] and in the UNet [4] architecture where a coupling between a feature encoding part and a reconstruction part facilitates the prediction of highly detailed output maps. With this work, we propose a parameter-efficient network structure that is well-suited for fast and accurate semantic classification of tissue patterns by combining paradigms from various architectures. We use a custom histological dataset to benchmark the classification performance of our network in comparison to UNet and FCN. Afterwards, a trained network is applied to a SMT dataset to highlight the relevance of the extracted tissue parameters.

## RESULTS

The network performance is evaluated in a cross-validation setting. Results for the accuracy, processing time and memory consumption are compared. Furthermore, we present prediction samples to show basic properties of the predictions. For the SMT setting, we apply the HistoNet processing pipeline and compare the performance after an expert verification step. Visualizations are given to indicate the descriptiveness of the features and cross-validation results for the diagnostic decision support are presented.

### Tissue classification performance

#### Evaluation

**Figure 1:**
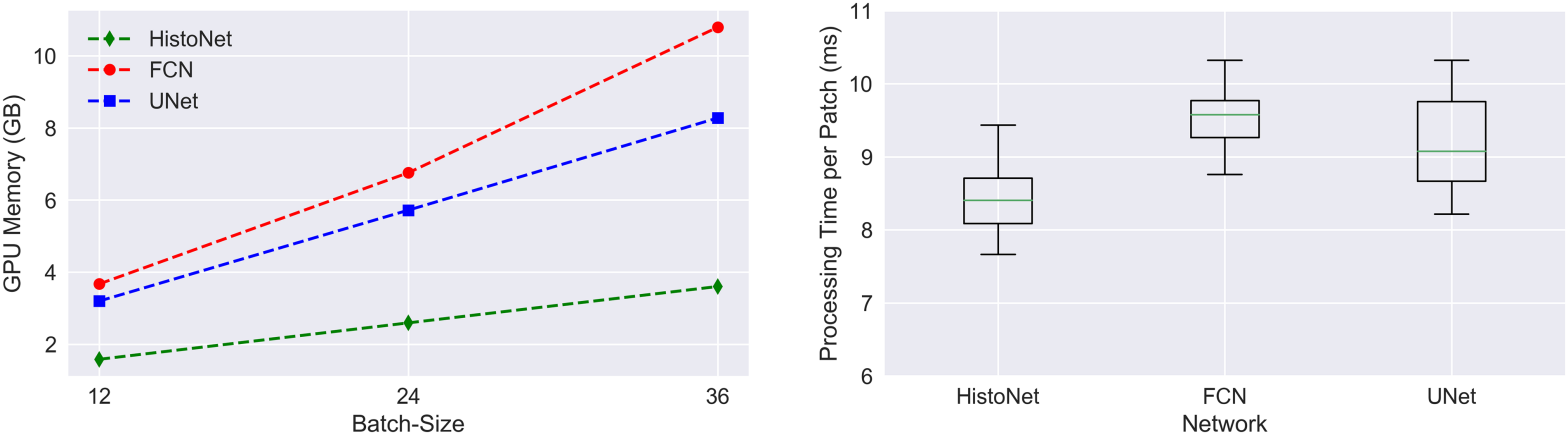
Memory consumption and processing time per image patch. Measured on an Nvidia Titan X (Pascal) GPU Device.

We refer to Table 1 for an overview of the classification performances. All architectures achieve a gain of approximately 28% compared to a baseline experiment with a classical feature pipeline. The respective F1-scores lie between 82% and 84%. In terms of computational time, see Figure 1, the networks are very similar regarding the required time per image, with a slight advantage for the presented HistoNet architecture. However, significant differences exist with respect to the memory requirement of the network types. While these differences are not necessarily relevant in research, they may be crucial in deployment, since extensive memory consumption can strongly limit the achievable bandwidth in practice. Furthermore, the built-in option in the proposed architecture to draw multiple samples to increase the accuracy and identify areas of uncertainty is a very desirable feature. For that reason and with no clear performance advantages of the competing architectures, we conduct later experiments using our HistoNet model.

**Table 1:** Summarized 5-fold cross-validation results of the tissue classification problem with eight distinct classes

Algorithm	Precision (%)	Recall (%)	F1-Score (%)
**Baseline**	58.0 ± 4.9	55.6 ± 5.3	55.0 ± 5.3
**FCN**	84.0 ± 1.3	84.0 ± 1.3	84.0 ± 1.3
**UNet**	82.2 ± 2.1	82.8 ± 1.8	82.0 ± 2.0
**Histonet**	83.4 ± 1.3	82.8 ± 1.0	83.0 ± 1.2

**Figure 2:**
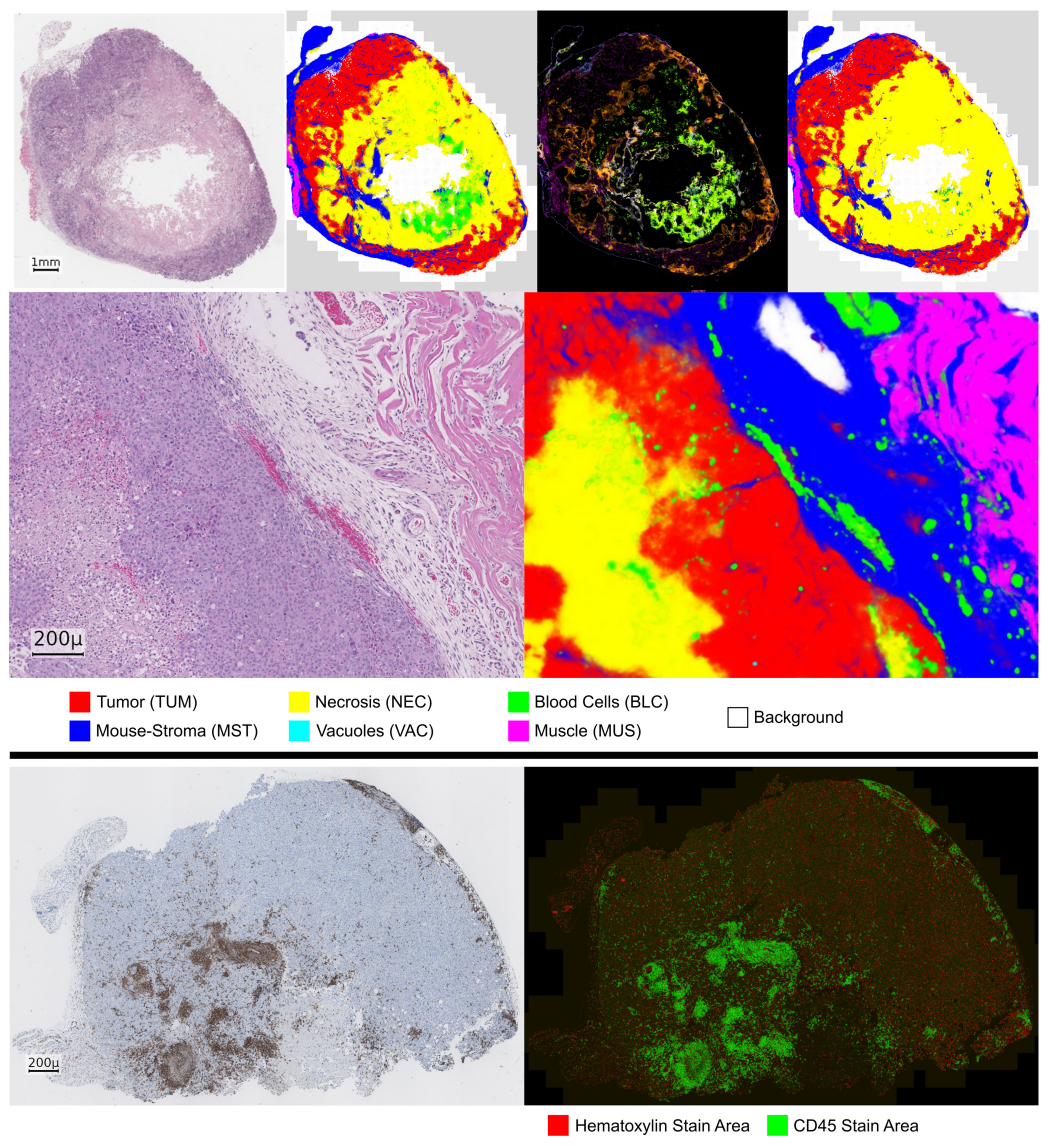
Prediction of the HistoNet architecture on an input sample (NSCLC PDX). Top, from left to right: input slide, prediction average map, variance map, corrected tissue map. Middle: detail view of a different slide (NSCLC PDX) with input (left) and prediction average map (right). In the variance map, light green as a mixture of green and yellow, corresponds to a confusion of BLC and NEC class. Bottom: CD45 example (left, NSCLC PDX) with corresponding stain color decomposition (right).

**Figure 3:**
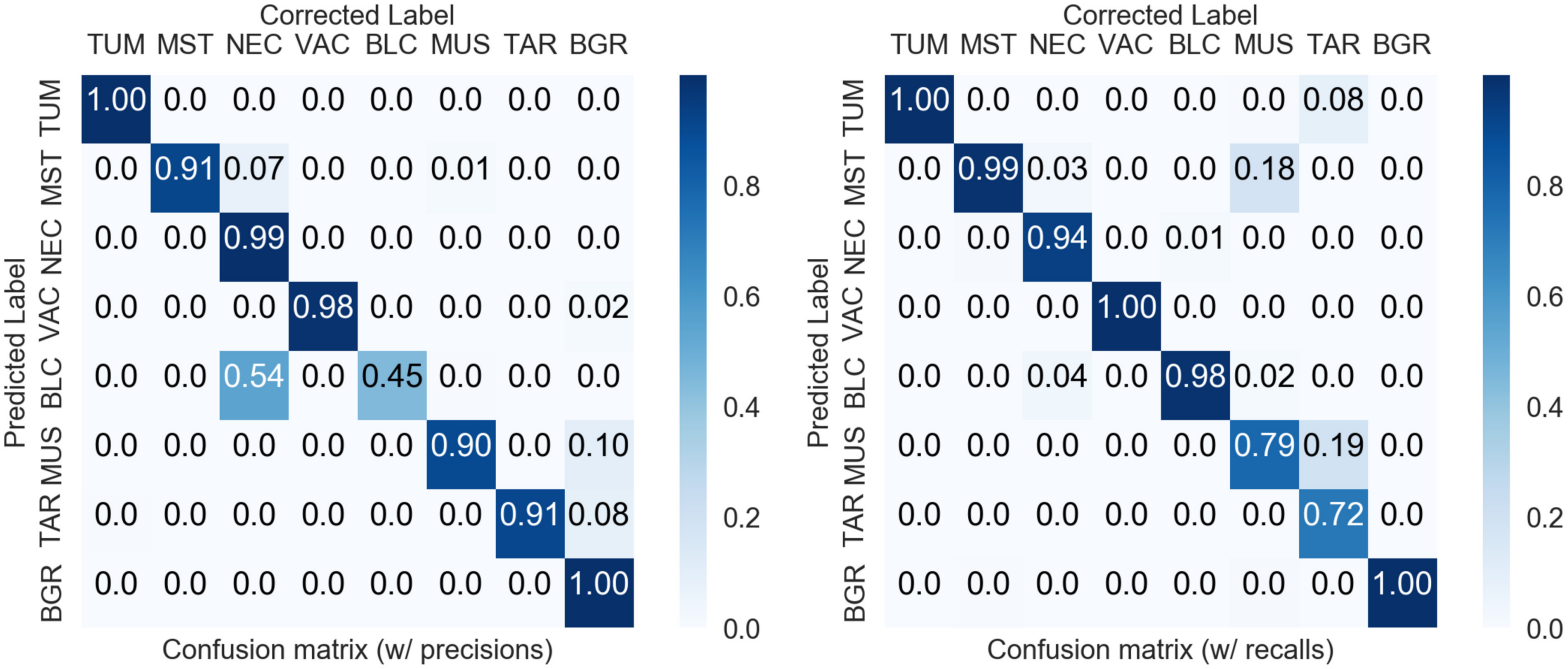
Confusion matrices of the manual corrections. Right: normalized to precision values. Left: normalized to recall values. Overall accuracy 98.3% (imbalanced), F1-score 89.4% (balanced).

From visual inspection of the predicted tissue maps in Figure 2, we observe that even in difficult cases a majority of the tissue is labeled correctly. A variance floor is usually present at the boundaries of tissues, particularly between tumor (TUM) and mouse-stroma (MST), which is to be expected. Between the classes bloodvessels/-cells (BLC) and necrosis (NEC) a more systematic confusion is present, see the examples in Figure 2 and the confusion matrices in Figure 3. The detail view reveals that in several cases even very fine stroma structures are detected. Furthermore, despite the strong underrepresentation in the data, blood-vessels in the stroma region are well segmented beyond our expectation.

### Single-mouse-trial analysis

In the SMT analysis, we apply the trained network to a large histological dataset and verify the use of the network by formulating a hypothetical diagnosis support problem. This setup represents a screening of different tumors and treatments. We compute meta-features from the predicted tissue-maps and CD45 images and analyze their distribution with respect to the expert’s diagnostic decision on the success of the treatment.

#### Visual inspection and error correction

As the neural network predicts human-interpretable maps of the tissue, a verification and correction step is conducted. Herein, the prediction variance map can be used as a guideline to identify areas of uncertain decisions. The inspection aims to correct large areas of mislabeled tissue to prevent error-propagation to the subsequent analysis, while we accept natural local uncertainties such as the tissue transitions between tumor – necrosis or tumor – mouse-stroma. Figure 3 shows confusion matrices comparing the network predictions to the corrected tissue maps. Most confusions happen between the classes BLC – NEC due to hemorrhagic areas and between mouse-stroma and necrosis due to residual fiber-structures in necrotic areas. As this confusion occurred systematically, this enabled us to correct the BLC – NEC confusion mostly algorithmically utilizing the variance map of the predictions by relabeling the areas of variance to the NEC class, see Materials and Methods. In total, only 1.7% of the predicted pixels needed correction, i.e. in 98.3% of the area the default predictions were accepted by the expert. Additionally, as the classification task is an imbalanced problem, it is worth considering a F1-scoring with equal weight per class and this measure results in 89.4%. Regarding the CD45 data, only one of 71 instances required a minor correction due to a stain-artifact. Because of the consistent labeling, the verification and correction of the 71 H&E and CD45 image pairs took less than six hours.

#### Visualizations of reference 2D feature spaces

**Figure 4:**
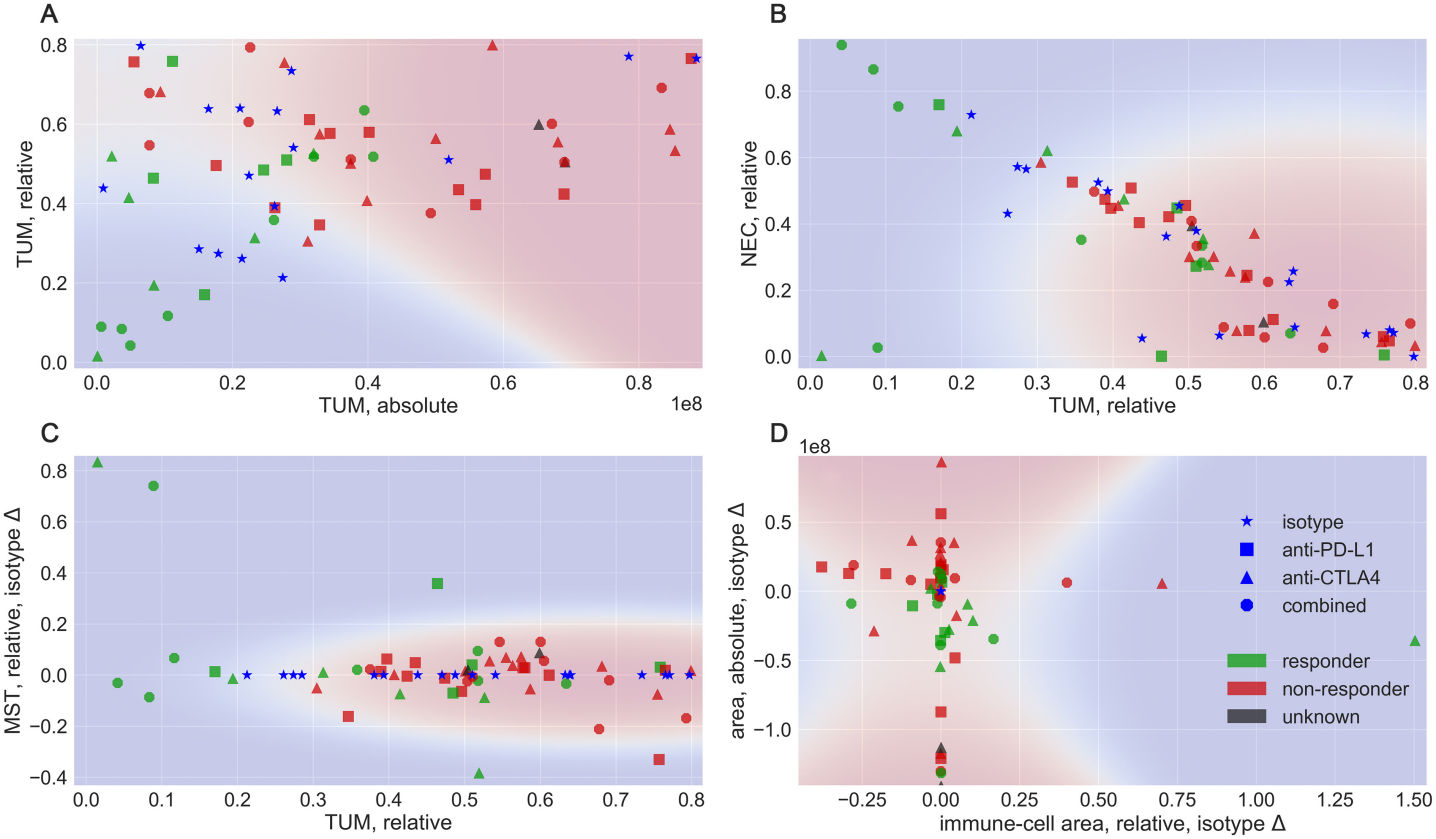
Examples of different feature combinations. Colors denote the response to treatment, in blue: isotype, green: responder and red: non-responder. Shapes denote the treatment type, as star: isotype, square: PD-L1 blocker, triangle: CTLA4 blocker and circle: combined treatment. The background colors indicate a probabilistic assignment by a Naive Bayes Classifier.

From the corrected prediction, we compute tissue meta-features according to the equations given in the Materials and Methods section. These features branch into the categories of absolute, relative, and isotype-difference features. In a first analysis, we visualize selected feature combinations in 2D, shown in Figure 4. Measures of the isotype models, denoted by the blue star symbol are plotted for reference. Additionally, we display a probabilistic assignment according to a Naive Bayes Classifier [5, 6] to indicate responsive (blue) and non-responsive (red) regions in the feature spaces, using a baseline classifier. Two of the strongest features, the absolute tumor (TUM) area vs. the relative TUM area in Figure 4A already separate a large portion of the samples. Particularly, the absolute TUM area (

x−axis)

indicates that a hard threshold between responder and non-responder models exists at approximately

$$0.45\times 10^{8}$$

px. Tumor areas larger than this threshold are all non-responsive to the treatment in this data.

The relative TUM area is not as distinctive but shares the property that tissues with less TUM content are more likely to be responsive. Consequently, a machine-learning boundary would separate the classes nearly linear along the image diagonal. An interesting dependency between the tumor- and necrosis-fraction of the tissue is illustrated in Figure 4B, where we observe an anti-proportional dependency between the relative TUM and NEC tissue. Herein, tumors with a high fraction of necrotic tissue and a low fraction of tumor are likely to have responded to the treatment, as the necrosis can be explained as decay of tumor tissue. Both constellations are good examples for the meta-features to reflect an intuition about biomedical dependencies. In Figure 4C, feature characteristics that measure the difference to the isotype, in this case for the stroma class are shown. Necessarily, the isotypes do not deviate from themselves and are therefore located on the

x−axis

(y=0)

in this plot. The computed decision boundary interprets large changes in the stroma tissue as a characteristic of responding models. Two isotype difference features are shown in Figure 4D, with the change in CD45 positive fraction vs. the change in overall tissue area. All isotypes collapse into the origin in this plot and as a more general observation, tumors with an increase in the overall area and a decrease in CD45 cells are likely non-responsive, while models with a decrease in the overall area and changes in CD45 cells are considered likely responsive by the Naive Bayes model. Additional visualizations are provided in the Supplementary Figures 2 and 3. As 2D visualizations represent low-dimensional subspaces, they cannot be expected to cover the complexity of the relations in the data completely. In the following, we evaluate the performance in a classification task using a high-dimensional feature space, to obtain an estimate for a potential application scenario.

#### SMT decision support scenario

Data available for this scenario is relatively limited for ethical reasons, as each sample corresponds to a sacrificed animal in addition to the isotypes. With 51 annotated samples for the decision-learning, we opt for established statistical methods that can handle a low number of samples. We estimate of the performance of the proposed processing pipeline in a decision support scenario, by reformulating the classification problem as a generalization task using a subset of the samples for learning and the remaining samples for inference and evaluation. We iterate these data splits ten times, such that each sample served in both roles and such that the individual splits approximate the true distribution of responders and non-responders, i.e. we perform a 10-fold cross-validation with stratified folds. In Table 2 we show the classification results for different classifiers [5, 7, 8]. The peak performance using a five Nearest-Neighbors Classifier and Manhattan-Distances achieves an average accuracy [7] of 84.2% and an average AUC-ROC [7] of 86.7%. Herein, we deploy a feature combination of absolute tumor area, relative tumor area, relative stroma area, and isotype-differences in the necrosis, CD45 area and overall area.

**Table 2:** Results of a 10-fold cross-validation classifying responding and non-responding patient-derived xenograft models

Classifier Model	Accuracy (%)	AUC-ROC (%)
**Baseline, Educated Guess**	59.2	59.6
**Baseline, Naïve Bayes [5, 6]**	70.8	76.7
**RBF-SVM [7, 19]**	76.8	83.3
**Linear SVM [20, 28]**	78.5	83.8
**Logistic Regression [7]**	78.2	84.2
**5-NN, Euclidean [5, 7, 8]**	76.8	83.2
**5-NN, Manhattan [5, 7, 8]**	**84.2**	**86.7**

The educated guess baseline always predicts non-responsive according to the data distribution.

## DISCUSSION

In this study, we present and evaluate a deep learning setup for the classification of PDX NSCLC tissue. It is demonstrated how interpretable network outputs – maps of the predicted tissue classes and prediction confidence – can be applied to an analysis of a large histological dataset. Focusing on remaining challenges regarding a tissue-classification with fixed ground-truth, roughly 17% of the pixels are still predicted inaccurately, creating a need for manual supervision prior to subsequent processing steps. For the stroma class, we observed that the prediction of fine fiber-like structures is still prone to misclassification. Typically, the network predicts the surrounding tumor class instead which creates a small bias that likely is negligible, in practice. Another source of confusion arises from less represented classes: vacuoles, blood vessels/cells and muscle. Vacuoles are often labeled as necrosis and vice-versa, a confusion that in some cases is uncritical and strongly depends on the definition of both structures, since advanced necrotic processes leave nothing but a diffuse plasma behind, which strongly matches the appearance of large vacuoles. Vice-versa, some of the larger annotated vacuoles may actually be the result of necrotic tissue decomposition. Furthermore, a confusion between blood-cells and necrosis may occur as a result of often co-located hemorrhagic areas. Retrospectively, the labeling of blood-vessels and hemorrhagic areas together in the blood-cells/vessels class likely caused this systematic misclassification. A correction would provide the characterization of the blood-vessel count, size and distribution as additional interesting biomarker for the tumor-micro-environment. Muscle tissue mostly appears as a very distinct structure, except for rare cases of inflammatory tissue from the necrosis or tumor class. Most of the confusions are likely the result of a strong underrepresentation and we see no further reason why these classes should not be recognized correctly, as the annotation database grows.

The characteristics of computed meta-features were inspected through visualization in low dimensional spaces and have shown to correspond to expected relations in the data. These feature spaces are only subspaces and cannot be expected to fully represent the complexity of the data. Higher dimensional models have a better potential to provide reliable solutions, however, they are not easy to visualize accurately - although manifold embedding techniques are sometimes applied for approximate impressions. For the purpose of reliable and reproducible results, a cross-validation is the more appropriate performance estimator, though.

In a hypothetical decision support setup, an accuracy of approx. 84% can be expected. Note that this result is to be taken as preliminary estimate due to two reasons: first, the relatively low number of samples, which we accept here for ethical reasons regarding animal sacrifice and the availability of tumor models, and second, the resulting over-engineering as this estimate uses a specific feature combination. Other suitable combinations operate with a performance difference between −3% and −6% which can still be considered as reasonable outcome.

In addition to the experiments describes here, we attempted to distinguish naturally occurring necrosis from treatment induced necrosis via textural image features – with negative result. Details are provided in the supplementary material along with a visualization of the texture feature embeddings, see Supplementary Figure 1.

While digital pathology algorithms for lung-cancer WSIs are not deeply covered in literature, there is a variety of publications regarding breast cancer detection [9], grading [10, 11], and epithelial vs. stroma classification [12]. Besides the differences in organ, technical deviations exist, as [9] solves a binary classification problem (versus our eight-class problem) and [10, 11] work with ordinal data. Similar to our approach, [12] deploys a two-step processing involving deep learning feature generation and conventional classification. All four references use patchwise classification instead of semantic annotation, effectively limiting the spatial resolution [10] or resulting in a trade-off between network parameters and computational speed [9].

## MATERIALS AND METHODS

### Tissue classification dataset

**Figure 5:**
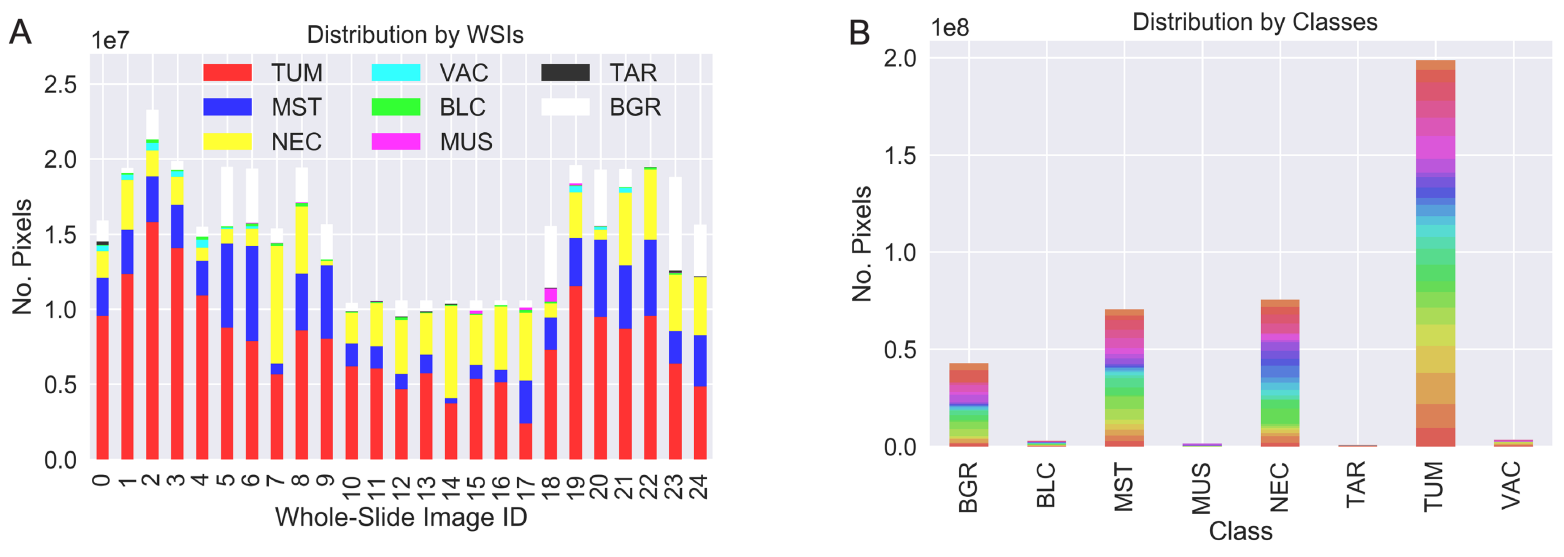
Two visualizations of the dataset distribution focused on the contribution from each WSI (**A**) and the class distribution (**B**). Colors in (B) represent the WSI origin. While we obtain a good balance of the labeled data per slide (A), the class-distribution (B) leads to a very imbalanced machine-learning task.

For the training and evaluation of the network performance, we labeled data comprising six distinct tissue classes: tumor (TUM), stroma/connective tissue (MST), necrosis (NEC), blood-cells/-vessels/inbleeding (BLC), vacuoles (VAC) and muscle (MUS) plus an additional class of technical-artifacts (TAR) and a background (BGR) class. The images are extracts from 25 different hematoxylin and eosin (H&E) stained whole-slide images showing lung-cancer and breast-cancer tumors grown in patient-derived xenograft models. Note that despite the differences in tumor types, the tissue classes remain quite similar in appearance. Biomedical experts annotated representative regions-of-interest, each showing different combinations of tissues at a resolution of 2 µm/px. Figure 5A shows the distribution of annotated pixels per slide and indicates their internal class distribution. An example of the annotation quality is given in Figure 5B.

#### Preprocessing

We perform stain-normalization using the Reinhard method [13] to center the color distribution of all slides. However, during training, the stain-colors were augmented along the principal color components, computed from the distribution across all the available data. Thus, the network sees the same image patch in different artificial stain variations during each training iteration. Additional augmentations were implemented with random translation, flipping and rotation.

#### Baseline experiment

Classical solutions based on texture and color features have been proposed to address multi-class problems in histology [14, 15]. Therefore, as a baseline for our experiments, we run a classical pipeline with statistical moments on RGB channels, greylevel co-occurrence features [16], local-binary patterns [17] and Tamura texture features [18] combined with different classification algorithms: support-vector machine [5, 19, 20] with various kernels and a random forest [5, 8, 14]. The ground truth for this patchwise setup was sampled from the semantic annotations and balanced among the different classes. The best configuration in terms of a weighted F1-score is a support-vector classifier with RBF kernel deploying the complete set of features.

### Network architecture

In this section, we explain our motivation for designing the custom classification network architecture. Both reference architectures FCN and UNet have a comparable contracting path built from blocks following a double convolution, nonlinearity, pooling pattern for the feature generation. Their main difference lies in the expanding network path that recomposes a detailed multidimensional output. FCN proposes an upsampling operation from feature to output dimension followed by concatenation and a few classification layers, whereas UNet suggests iterative feature-upsampling, -conatenation, and -convolution cycles until the input resolution is reached. Because of the upsampling to full output size of each contributing network block, FCN suffers severely from memory constraints in practice, resulting in small batch-sizes for training and small region-of-interest sizes in application. However, there are only relatively few parameters connecting the features to the actual classification output. In contrast, UNet uses about twice the number of parameters for reconstruction in the additional convolution steps, but drastically reduces the memory consumption through its iterative upsampling strategy. Architectures that pay attention to efficiency specifically deploy 1×1 convolutions to compress the number of channels and filter redundant feature responses into a more compact representation. Convolutions with spatial context, i.e. 3×3 Semantic Tissue Segmentation 3 and larger, are preferred to operate in a compressed feature space to save parameters. For example, ResNet [21] controls the number of parameters with its bottleneck-pattern: 1. a 1×1 convolution decreases the number of features, followed by 2. one or more 3×3 convolution layers and 3. a 1×1 convolution for expanding the features to the original number of channels, which is necessary to compute the residual. Thus, the spatial convolution is performed in a compressed feature space, reducing the number of parameters and constraining the data-flow.

In their original implementations, neither FCN nor UNet had access to the residual learning concept [21] in which information can bypass each network block, as their function represents a deviation from the data flow through the network. Residual blocks enable the training of very deep architectures, since the concept of learning many small deviations from a mean data flow alleviates the problem of exploding or vanishing gradients. While the skip connections in UNet may have a similar effect, note that an entire subnetwork is by-passed instead of a single block. At least in theory, the residual activations should be of low mean and variance, and regularization by Batch-Normalization [22] has often been applied to ensure this. Recently, a non-linearity with self-normalizing properties (SELU) [23] has been proposed, which together with $L_{2}$-Regularization of the network weights, contributes another way to ensure a reasonably bounded distribution on the residual activations, but without the additional parameters of a Batch-Normalization layer. SELU activations depend on a specific version of Dropout [23, 24]. Therefore, with the inherent presence of Dropout layers we have an optional source of randomness during inference and can utilize this to sample multiple predictions for the same patch. Instead of a single prediction, a mean and variance tissue map are computed. Specifically, the variance map helps to identify areas of uncertainty, which are located in areas where the Dropout of individual features leads to a change in prediction. It has been shown that this uncertainty correlates with the confusion of classes and may be used to optimize the labeling procedure [25].

In summary, the following design choices motivate our architecture:

- preferring residual blocks to facilitate a good gradient flow
- deploying bottleneck patterns for parameter efficiency
- inserting additional compression layers to balance feature concatenations
- controlling parameter spaces by regularization and self-normalizing non-linearities
- applying multi-objective learning to support domain-oriented learning
- optionally utilizing (Alpha-)Dropout during inference to sample multiple predictions

**Figure 6:**
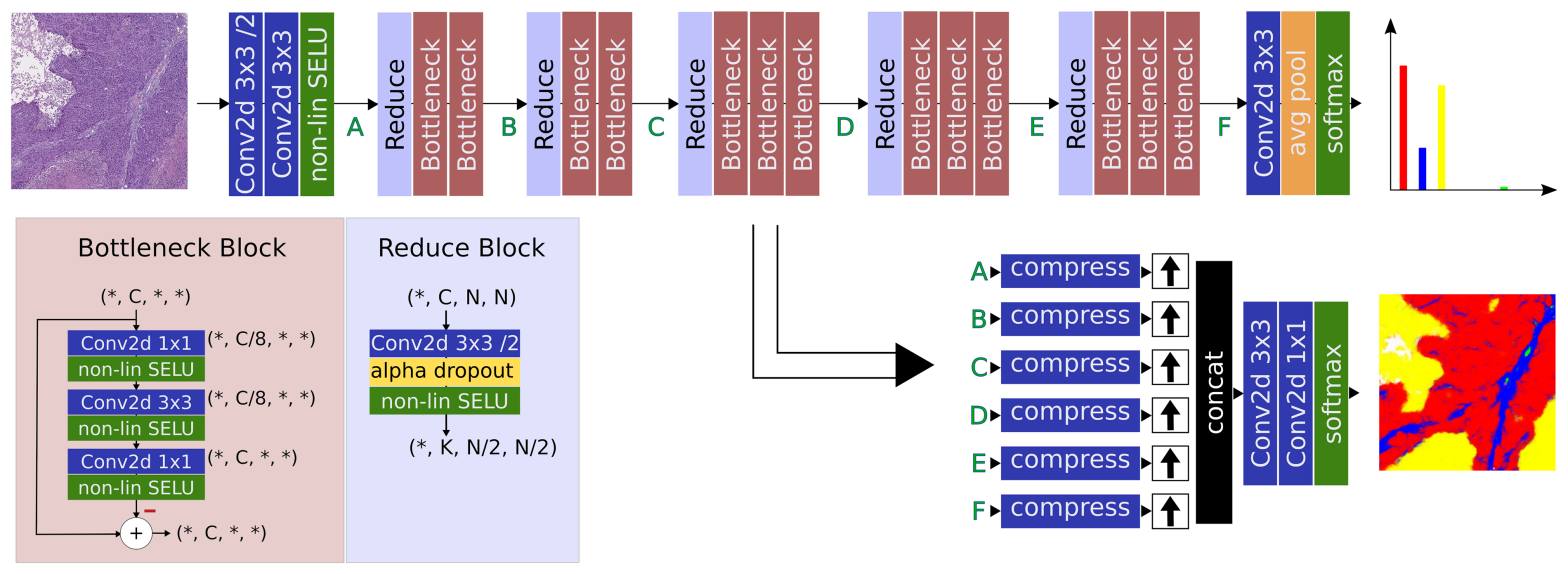
Overview of the proposed pipeline. A ResNet-inspired feature generation path is used together with a simple reconstruction network using 1×1 convolutions as compression for feature balancing and reduced memory consumption.

Following the above paradigms, we define the block structures in our architecture as a series of bottleneck blocks, as displayed in Figure 6.

The basic bottleneck block performs the compress – convolve – expand pattern in a residual function on the data path concluded by the self-normalizing SELU operation, while a reduce block contributes a strided convolution to decrease the spatial dimension. As a requirement of the SELU non-linearities, the regularizing dropout function is implemented as Alpha Dropout [23] inside the reduction blocks.

The network has two output paths. Semantic classification is achieved by compressing all feature levels via 1×1 convolutions followed by bilinear upsampling and concatenation right before the classifier, similar to the FCN architecture. The number of compression channels on each feature level is used to balance between coarse and detailed information, as indicated in the lower part of Figure 6. This output predicts a map of probabilities for each tissue class and is trained with a combination of categorical cross-entropy (CCE) loss and dice-distance loss (DDL). A second feature output of the last layer is implemented to directly predict the normalized tissue distribution in the input patch, as seen in the upper part in Figure 6. Herein, the normalization is implicitly achieved utilizing a Softmax non-linearity at the output. In conjunction with the true distribution, which can easily be computed on-the-fly from the semantic annotations, this output contributes a mean-square-error (MSE) loss. Thus, the final loss is the sum of the above loss contributions

$$L=L_{\text{CCE}}+L_{\text{DDL}}+L_{\text{MSE}},$$

which realize the paradigm of learning from multiple objectives. In the following, the proposed network is referred to as HistoNet.

#### Training and evaluation process

All networks pass a fivefold cross-validation (CV) scheme with fixed splits to ensure equal conditions for each algorithm. We chose the splits manually to balance the occurrence of rare classes among all folds. Furthermore, it is ensured that images from a particular patient or WSI are exclusive to a single split, i.e. test and training data are strictly separated with respect to the patients. As optimizer, we deployed Adam [26], with a learning rate of $5\times 10^{-4}$ and weight-decay $10^{-6}$.

### Single-mouse-trial analysis workflow

#### Single-mouse-trial dataset

An initial dataset of single-mouse-trials comprises 71 whole-slide images of hematoxylin and eosin (H&E) stained PDX non-small cell lung-cancer (NSCLC) tissue. The data is further subdivided into 17 groups in which the same tumor model provides each of the following four treatments: 1. isotype, 2. anti-PD-L1, 3. anti-CTLA4 and 4. combined anti-PD-L1 + anti-CTLA4 – providing 68 slides. The remaining three slides are an additional isotype and two anti-PD-L1 treatments for one of the tumors. This set is referred to as H&E data and is used for the tissue-class prediction. A second set of 71 whole-slide images consists of immunohistochemical staining using an anti-human CD45 antibody for detection and diaminobenzidine for staining the detected cells. This subset is referred to as *CD45 data* and is used for immune-response characterization. An expert labeled all 53 treated tumors as either *responding*, or *non-responding* to the treatment or in two cases *unknown*. Following the current standard workflow, the labeling decision is based on the recordings from flow-cytometric analysis, reference values of the stroma content (qPCR) for the different tumor models, the tumor-volume development during the experiment, and the observation of the histology images.

#### Processing

All WSI undergo a foreground selection [27] and computed tissue areas are sampled grid-wise at 10× objective-magnification such that the resulting patches overlap with 25%. The H&E patches are normalized [13] using the normalization parameters of their respective slide and segmented via the proposed HistoNet architecture. Using the stochastic classification approach, five predictions are sampled and we compute an average prediction map and a prediction variance map. From the average prediction map we compute the final predicted label per pixel as the class with the highest probability. At overlapping borders of extracted patches, bilinear interpolation is applied to merge the predictions into a tissue map and a corresponding variance map. A color-coding with the correspondences red: TUM, blue: MST, yellow: NEC, cyan: VAC, magenta: MUS and green: BLC is used for the six main classes, while TAR and BGR are mapped to black and white, respectively. Using a principal component analysis, the CD45 patches are destained into hemtoxylin-blue and diaminobencidine-brown component to provide a measure for the immune-interaction between the tumor and host immune-system.

### Automated correction of the systematic BLC – NEC confusion

As mentioned, a systematic confusion of the NEC – BLC class was observed in the variance map. An automated correction selects areas of light-green in the variance map and relabels the selection to the necrosis class in the predicted tissue map. For the color channels $V_{r},\;V_{g},\;V_{b}$, of the variance map V, the selection is computed using the red to green ratio $r=\frac{V_{r}}{V_{g}}\in \left(0.35,\;0.7\right)$ while $V_{b}$ is required to be close to zero. The range is chosen empirically, but with the knowledge that a 50% mixture of yellow and green, corresponding to the confusion of BLC – NEC, would result in r=0.5 and $V_{b}=0$, exactly. A Gaussian Blur (σ=5 px) is used to blend the selection smoothly into the tissue map.

### Computation of tissue meta-features

While the learned mapping from raw image data to tissue classes is a function that is hard to analyze, the computed tissue maps are a concept which is easy to understand and can be efficiently verified by experts as an intermediate step. After verification, the tissue maps are used to compute meta-features to extract clinically/research relevant parameters of the tumor and its composition.

A first set of features simply summarizes the absolute area of a tissue class with label-id i∈{0,1,…,L−1}, given L distinct classes

$$f_{i}^{\left(abs\right)}=\sum_{x,y}\begin{array}{cc} I\left(x,y,i\right), & I\left(x,y,i\right) \end{array}=\{\begin{array}{cc} 1 & \text{if }P\left(x,y\right)=i\\ 0 & \text{else} \end{array}$$

where P(x,y) is the prediction (or average prediction in the probabilistic case) of the network. The tissue area is then defined as summation of all major classes, omitting artifacts and background

$$A=\sum_{i}f_{i}$$

Relative measures of the tissue classes are computed as ratio of pixels per class divided by the tissue area.

$$f_{i}^{\left(\mathrm{rel}\right)}=\frac{f_{i}^{\left(\mathrm{abs}\right)}}{A}$$

Furthermore, we can relate the absolute or relative measures of the tissue to the corresponding isotype, by subtracting the respective isotype measure. Thus, this type of measure characterizes deviations in a feature under treatment conditions

$$\Delta f_{i}^{*}=f_{i}^{*}-f_{i,\text{ isotype}}^{*},$$

wherein ^*^ indicates either relative (rel) or absolute (abs) features. These features mainly target an analysis of H&E-stained tissue. However, immunohistochemical stains can be treated in a similar way by measuring the positive class, typically in diaminobenzidine brown, and by normalizing it with the number of pixels of the counter-stain, typically hematoxlyin-blue. Note that this feature, as well as the total area A, can be related to the isotype in the fashion of $\Delta f_{i}^{*}$ as well.

### Diagnostic decision support

A selection of the proposed features is used to support the clinical/research relevant decision if the TME is influenced by the treatment. Applicable features are concatenated into a vector and are used in conjunction to learn the difference between the parameters of responsive and non-responsive tumor models. Furthermore, we utilize two-dimensional subspaces of selected features to visualize decision boundaries of machine learning algorithms in the dataset. Since the individual features extend to rather different numeric ranges, we apply a min-max-normalization before the training of a Naive Bayes Classifier [8] for the visualization, or in case of the evaluation in later experiments a K-Nearest-Neighbor (KNN) classifier [7]. For the visualization, we decided for a Naïve Bayes approach as its decision boundaries have a simple structure and an inherently probabilistic nature resulting in smooth transitions between the class areas. In contrast, visualizations of a KNN algorithm (with K>1) tend to result in decision boundaries, or probability plateaus, with the data rendered close-to, but not inside, the respective class area which appears counter-intuitive despite very good results in a cross-validation.

## CONCLUSIONS

The proposed deep learning pipeline competes with state-of-the-art architectures at a F1-score of approximately 83% on a histological dataset. Differences between the networks are visible in the computational efficiency regarding processing time and memory consumption and correspond to the design choices as expected. Sampling multiple predictions at inference time using dropout mechanics provides relevant insights to the network behavior and options to compensate the observed systematic BLC – NEC confusion semi-automatically. In practice, the relative tissue area requiring correction was rather low (approx. 2%) which might indicate that the network already operates close to an inter-observer-variability boundary.

With a high relevance for research and clinical applications, the proposed image analysis pipeline facilitates the quantification of important biomedical markers in a non-destructive and therefore reproducible experimental setup. Deep learning features own a reputation of being hard to interpret. We partially circumvent this by computing an intermediate tissue map as a human-understandable and verifiable source of meta-features. These meta-features have shown to characterize properties of TMEs realistically and provide useful predictions in a machine-learning based decision support setting.

Future co-registration of H&E and IHC images would enable region-specific features measuring the co-localization of immune cells and tissue classes as a promising application case for this analysis.

## SUPPLEMENTARY MATERIALS



## Abbreviations

**Histology**
SMT: Single-Mouse-Trial
TME: Tumor-Micro-Environment
PDX: Patient-Derived Xenograft
NSCLC: Non-Small-Cell Lung-Cancer
WSI: Whole-Slide Image
CD45: Protein Tyrosine Phosphatase, Receptor Type C
H&E: Hematoxylin and Eosin
CTLA4: Cytotoxic T-Lymphocyte-Associated Protein 4
PD-L1: Programmed Death-Ligand 1
QPCR: Quantitative Polymerase Chain Reaction.

**Class labels**
TUM: Tumor (colorized in red)
MST: Mouse Stroma (colorized in blue)
NEC: Necrosis (colorized in yellow)
VAC: Vacuole (colorized in cyan)
MUS: Muscle (colorized in magenta)
BLC: Blood-Cell/Vessel (colorized in green)
TAR: Technical Artifact (colorized in black)
BGR: Background (colorized in white)

**Classifiers**
CV: Cross Validation
KNN: K-Nearest-Neighbors
SVM: Support-Vector Machine
RBF-SVM: Radial-Basis-Function-SVM
FCN: Fully Convolutional Network
SELU: Scaled-Exponential Linear Unit
CCE: Categorical Cross-Entropy
DDL: Dice-Distance Loss
MSE: Mean-Squared-Error
AUC-ROC: Area-Under-Curve-Receiver-Operator Characteristic
