# Supplementary material for: Semi-automated analysis of digital whole slides from humanized lung-cancer xenograft models for checkpoint inhibitor response prediction
Source: Oncotarget. 2019 Jul 16;10(44):4587–97. doi: 10.18632/oncotarget.27069 (PMC6642041; doi:10.18632/oncotarget.27069)
Supplement: Supplementary file 1 [file oncotarget-10-4587-s001.pdf]

Semi-automated analysis of digital whole slides from humanized lung-cancer xenograft models for checkpoint inhibitor response prediction

SUPPLEMENTARY MATERIALS

Distinction of preexisting and induced necrosis

During the review of the manuscript, the question was raised, whether our approach could distinguish between preexisting necrosis and induced necrosis. Preexisting necrosis would be the result of rapid tumor growth leading to insufficient vascularization, compression and thrombotic obstruction of vessels, while induced necrosis would be the result of treatment. In response, we conducted additional experiments that are described in this section. In essence, we tested if image features computed on necrosis patches can predict, if a model is an isotype or was treated. However, the result is negative: the distinction is not possible – at least with the evaluated image features.

Methods

From all necrotic regions, determined by the tissue maps, we extracted patches and generated features: classical Greylevel Co-occurance/Haralick features, Color-Histograms, and DenseNet features (using the ImageNet pretrained parameters).

Two evaluations were performed:

A. We computed TSNE-embeddings to visualize the high-dimensional feature spaces in 2D. As the examples given in the attached Figure 1 show, there is no clear separation in the Point-Clouds generated from Treated (green) and Isotype (red) samples. Since this is only a qualitative assessment, we proceeded to:

B. Evaluating the predictivity of the features for a treatment or isotype model. To this end, we tested different classifiers (Linear Discriminant Analysis, Quadratic Discriminant Analysis, Support-Vector Machine, K-Nearest-Neighbors) in a cross-validation across patients (=PDX models).

Experiment B iterates various options exploring optional feature standardization, dimensionality reduction and feature combinations. Additionally, we tested the features per-patch and after a per-model accumulation using mean and std across all necrosis patches of the respective model as new combined features.

For performance assessment we used Accuracy and AUC-ROC, as in the SMT decision support experiment.

As baseline performances it is reasonable to assume an educated guess performance of approximately 70% Accuracy. This matches the ratio of treatment samples to isotypes. The AUC-ROC measure considers the classes independently from their frequency and thus has a baseline of 50%.

Results

None of the configurations achieves a notable difference from the baseline performance.

This confirms the observation in experiment A (see Figure below) that none of the Embeddings show a separation of the two groups (isotypes vs. treated models).

Thus, we have to conclude that the image features are not predictive for preexisting and induced necrosis.

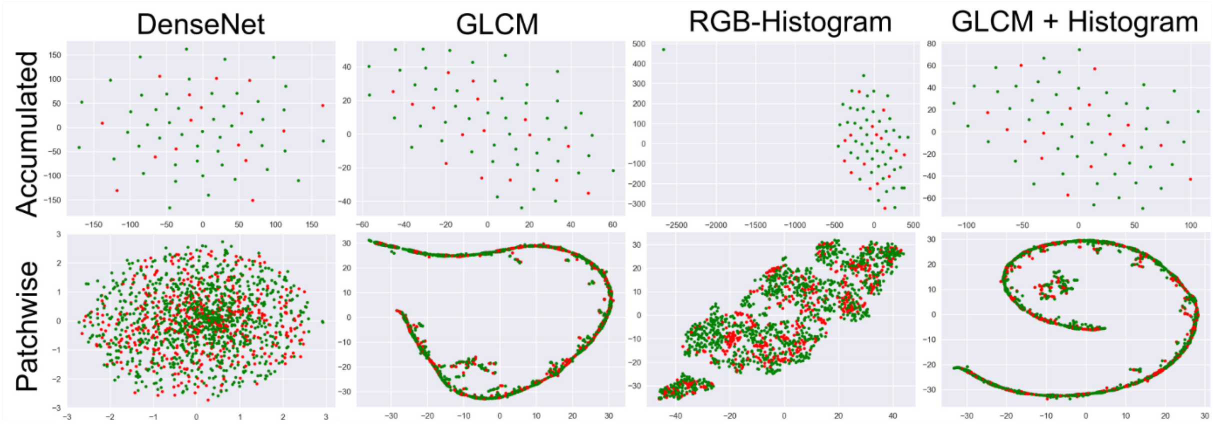

Supplementary Figure 1: Examples of the different embeddings. A clear separation of isotype models (green) and treated models (red) would mean an indication for predictivity, instead, the point clouds show a diffuse mixture of samples. The snake-like outer structure of the patchwise GLCM combinations is no exception regarding point distribution along the curve.

Additional 2D feature visualizations

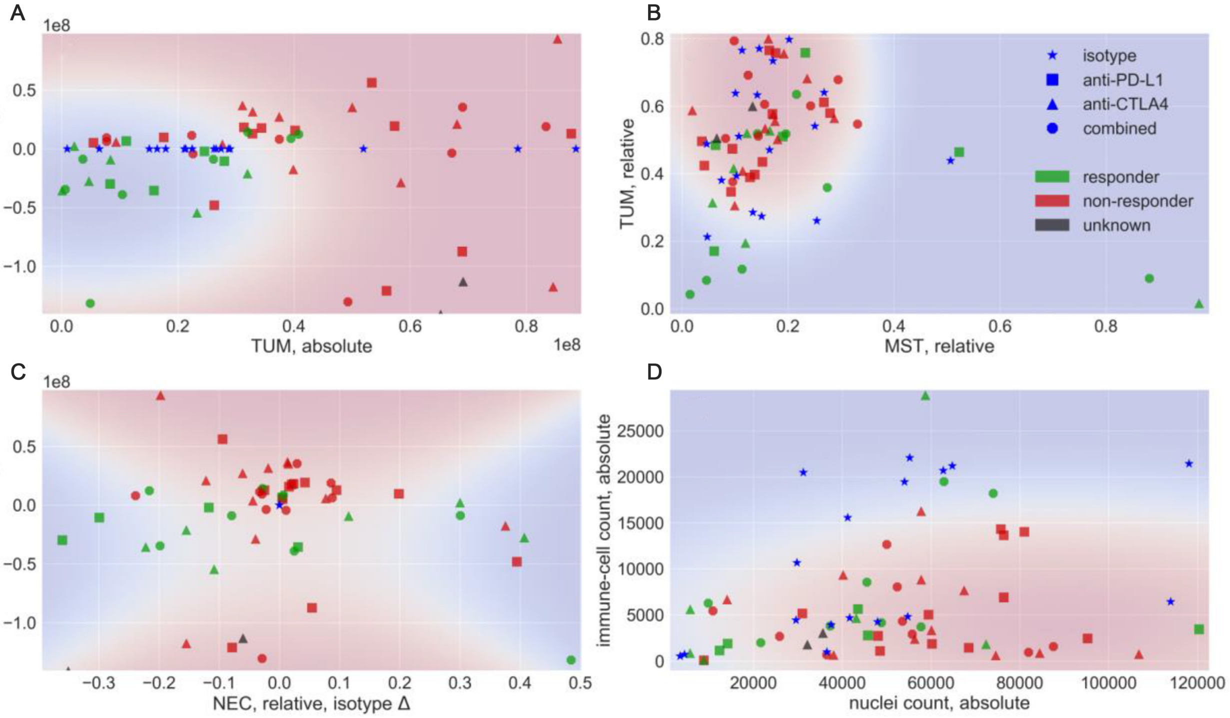

Supplementary Figure 2: Other feature constellations in 2D. The features in (**A–C**) are part of the proposed feature combination for the decision support scenario, while (**D**) shows the distribution of raw nuclei counts.

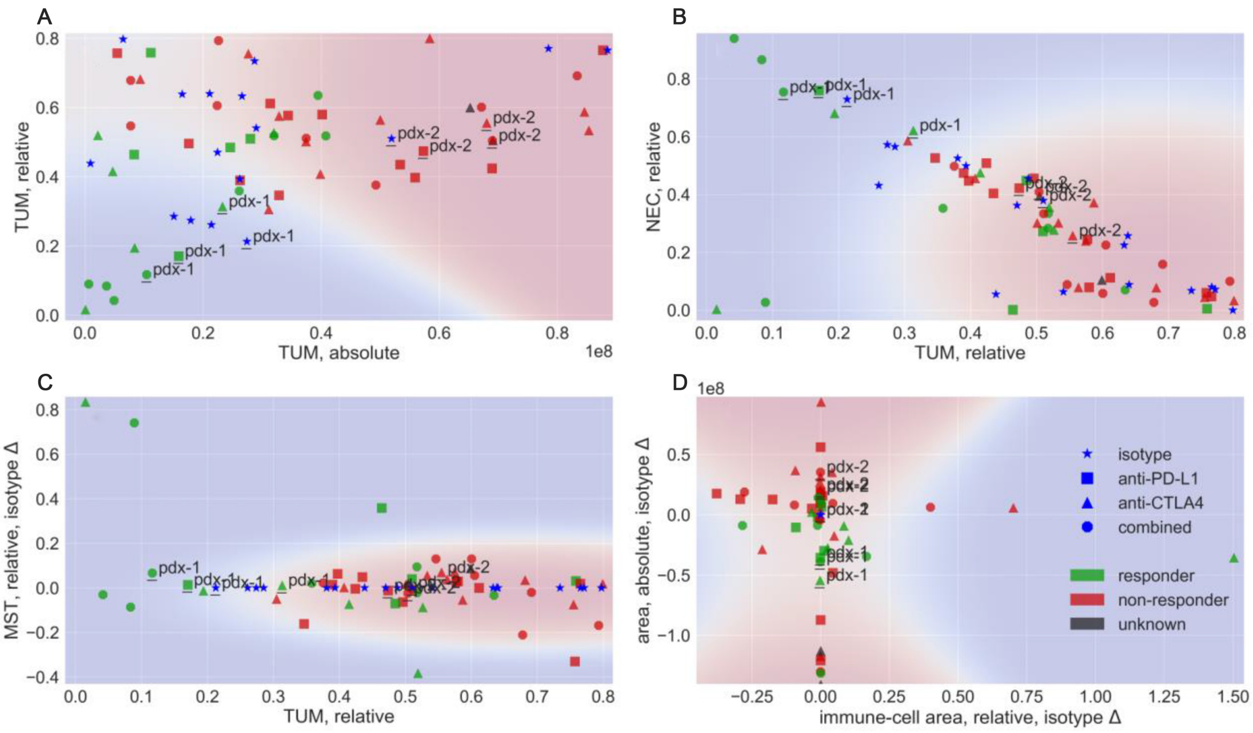

Supplementary Figure 3: Same constellations as in Figure 4 of the publication, but with additional labels indicating two tumor models (pdx-1 and pdx-2). The corresponding symbol is underlined to the left of the label. Despite the number of samples this can be used to indicate the experimental groups.
